# An Electrospun Preparation of the NC/GAP/Nano-LLM-105 Nanofiber and Its Properties

**DOI:** 10.3390/nano9060854

**Published:** 2019-06-04

**Authors:** Tingting Luo, Yi Wang, Hao Huang, Feifei Shang, Xiaolan Song

**Affiliations:** 1 School of Materials Science and Engineering, North University of China, Taiyuan 030051, China; luotingting1002@163.com; 2 China North Industries Group Corporation Limited, Beijing 100821, China; huanghao-xiang@163.com; 3 Teaching and Research Support Center, Army Academy of Armored Forces, Beijing 100072, China; feiniaozi@126.com; 4 School of Environment and Safety Engineering, North University of China, Taiyuan 030051, China; songxiaolan00@126.com

**Keywords:** electrospinning, NC/GAP/nano-LLM-105, thermolysis, energetic performance, sensitivity

## Abstract

In this work, an energetic composite fiber, in which 2,6-diamino-3,5-dinitropyrazine-1-oxide (LLM-105) nanoparticles intimately incorporated with a nitrocellulose/glycidyl azide polymer (NC/GAP) fiber, was prepared by the electrospinning method. The morphology and structure of the nanofiber was characterized by scanning electron microscopy (SEM), energy dispersive X-Ray (EDX), fourier transform infrared spectroscopy (IR), X-ray diffraction (XRD), X-ray photoelectron spectroscopy (XPS), and Brunauer–Emmett–Teller (BET). The nanofibers possessed a three-dimensional (3D) net structure and a large specific surface area. Thermal analysis, energetic performance, and sensitivities were investigated, and they were compared with NC/GAP and LLM-105 nanoparticles. The NC/GAP/nano-LLM-105 nanofibers show higher decomposition rates and lower decomposition temperatures. The NC/GAP/nano-LLM-105 decomposed to CO_2_, CO, H_2_O, N_2_O, and a few NO, -CH_2_O-, and -CH- fragments, in the thermal-infrared spectrometry online (TG-IR) measurement. The NC/GAP/nano-LLM-105 nanofibers demonstrated a higher standard specific impulse (*I_sp_*), a higher combustion chamber temperature (*T_c_*), and a higher specialty height (*H*_50_). The introduction of nano-LLM-105 in the NC/GAP matrix results in an improvement in energetic performance and safety.

## 1. Introduction

2,6-Diamino-3,5-dinitropyrazine-1-oxide (LLM-105) is an essential ingredient in many propellant and explosive formulas for its low sensitivity, high energy, high density, and excellent thermal stability [1,2]. The properties of low sensitivity and excellent thermal stability are attributed to the π-conjugated system. Due to the intense intramolecular hydrogen bonding, the LLM-105 possesses good compatibility with the common components of propellant and explosives, such as hexahydro-1,3,5-trinitro-1,3,5-triazine (RDX), nitrocellulose (NC), etc. [3,4,5,6]. Nanoscale LLM-105 has a superior energy release rate and a higher reaction rate, compared with conventional LLM-105 [7,8,9,10]. However, the agglomeration of nano-LLM-105 causes a decrease in performance and limits its application [11,12]. It is feasible that energetic matrix is utilized to support LLM-105 nanoparticles, which can effectively avoid agglomerating.

Electrospinning is a universal technology that is used to obtain multifarious nanocomposite [13,14,15,16]. The as-spun 3D nanofibers, with high specific surface areas and porosities are desired carrier for supporting nanoparticles [17,18,19]. However, the application of electrospinning technology in composite energetic materials is rarely performed [20]. For instance, nitrocellulose/aluminum-cupric oxide (NC/Al-CuO) and nitrocellulose/2,4,6,8,10,12-hexanitro-2,4,6,8,10,12-hexaazaisowurtzitane (NC/CL-20) nanofibers with high burning rates were obtained via electrospinning [21,22]. Li also fabricated nanoboron/nitrocellulose (B/NC) electrospun nanofibers with excellent thermostability [23]. Similarly, researchers selected single NC as electrospinning matrix. However, simplex NC has a relatively large viscosity, which affects the morphology of the nanofiber, and results in low loading of the nano-explosive. In addition, a high spinning voltage could generate electric sparks and bring great safety risks. The composite energetic matrix can compensate these deficiencies. GAP is a high-energy prepolymer with low viscosity and high density. Moreover, it has more flexible segments, a lower glass transition temperature (*T_g_*), and higher mechanical properties than NC. Currently, there is no report on using GAP as a matrix to load explosives with electrospinning [24,25,26]. In this work, ball milling nano-LLM-105 is assembled onto a NC/GAP composite matrix by electrospun technology, to create a new type of energetic 3D nanocomposite. In fact, it is not dangerous to prepare explosive materials by electrospinning and ball milling. This is because that the energetic material is very stable at a normal temperature and pressure, and under the protection of solvent. At this time, they are no different from inert materials. Further experiments suggest that the NC/GAP/nano-LLM-105 nanofibers possess lower sensitivity and remarkable thermal decomposition and energy performance, which makes the nanofibers have application potentials in the field of solid propellants.

## 2. Materials and Methods 

### 2.1. Materials

2,6-Diamino-3,5-dinitropyrazine-1-oxide (LLM-105) was provided by Gansu Yinguang Chemical Co., Ltd. (Baiyin city, Gansu province, P.R. China). Glycidyl azide polymer (GAP, Mn = 4000, hydroxyl value of 0.49 mmol·g^−1^) was obtained from the 42nd Institute of the Fourth Academy of China Aerosce Science and Technology Corporation. Nitrocellulose (NC, 12.6% N, industrial grade) was provided by Foshan Junyuan Chemical Co., Ltd. (Foshan city, Guangdong province, P.R. China). Ethanol (EtOH) and acetone were purchased from Tianjin Guangfu Chemical Co., Ltd. (Tianjin city, China).

### 2.2. Fabrication of Nanofibers

**Figure 1:**
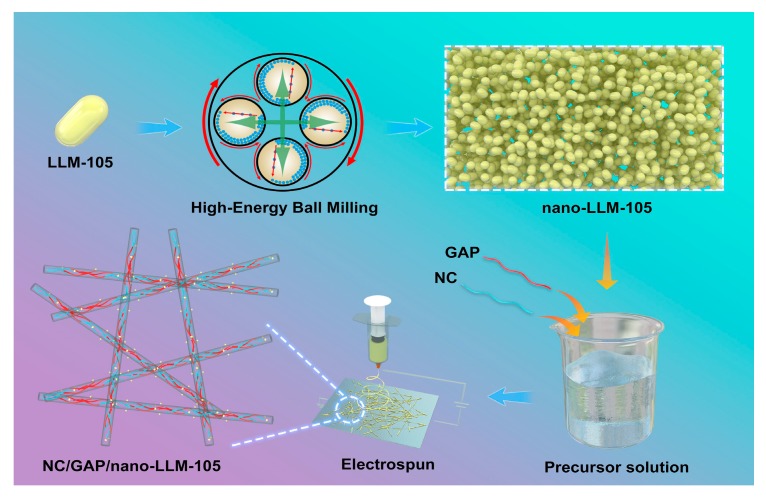
Sketch for the synthesis of the nitrocellulose/glycidyl azide polymer/nano 2,6-diamino-3,5-dinitropyrazine-1-oxide (NC/GAP/nano-LLM-105) composite nanofiber.

Firstly, nano-LLM-105 was prepared by the high-energy ball milling method. The ingredients, including 200 g balls, 6 g LLM-105, 60 mL deionized water, and 60 mL ethanol, are added into a mill jar. The four jars are sealed and immobilized on the ball mill. The mill rotates at 300 rpm for 6 hr. Then 0.3 g nano-LLM-105 is dissolved in 4.4 g acetone to get nano-LLM-105 suspension. Exactly 0.45 g NC and 0.45 g GAP were added into 4.4 g acetone to obtain a NC/GAP solution. The above-prepared nano-LLM-105 suspension and NC/GAP solutions were blended to obtain a NC/GAP/nano-LLM-105 precursor (12 wt %). The mass ratio of NC, GAP, and nano-LLM-105, respectively was set to 3:3:2. As a contrast, the NC/GAP precursor solution (12 wt %) was obtained by dissolving 0.6 g NC and 0.6 g GAP into 8.8 g acetone. The mass ratio of NC and GAP is set to 1:1. For the electrospinning process of these two nanofibers, the inner diameter of the stainless steel needle is 0.8 mm. The ambient humidity was controlled at 40–50%. The applied voltage was maintained at 12–18 kV. Additionally, the flow rate was fixed at 3–5 mL·hr^−1^. Aluminum foil was used to collect the fibers, which were placed 12 cm away from the needle. The preparation scheme is described in Figure 1.

### 2.3. Characterization

The analyses of SEM, EDS, IR, XRD, and XPS were performed in order to investigate the morphology and structure of NC/GAP, nano-LLM-105, and NC/GAP/nano-LLM-105. Scanning electron microscopy (SEM) was performed on a Hitachi SU8010. The diameters of particles and fibers were measured by Nano Measurer 1.2 software. X-ray diffraction (XRD) analysis was performed on a DX-2700 X-ray diffractometer (Hao yuan) with Cu Kα radiation. The IR spectrum was obtained on an infrared spectrometer (American Thermo Fisher Scientific Nicolet 6700). XPS was conducted with X-ray photoelectron spectroscopy (XPS) and a PHI5000 Versa-Probe (ULVAC-PHI). The BET measurements of NC/GAP and NC/GAP/nano-LLM-105 were performed, utilizing nitrogen adsorption with a Micromeritics ASAP 2010 instrument. Thermal analyses for NC/GAP, nano-LLM-105, and NC/GAP/nano-LLM-105 were conducted on a differential scanning calorimeter (DSC, TA Model Q600) at heating rates of 5, 10, 15, and 20 °C/min. thermal-infrared spectrometry online (TG-IR) analyses of NC/GAP and NC/GAP/nano-LLM-105 were performed on a thermal analyzer system (TG/DSC, Mettler Toledo) coupled with a Fourier transform infrared spectrometer in a nitrogen atmosphere. The temperature range that we considered was 50 °C to 400 °C. The impact sensitivity was tested by using HGZ-1 impact equipment. In each test, 25 drop tests were carried out to calculate the *H*_50_, and each portion was performed three times to obtain a mean value and a standard deviation.

## 3. Results and Discussion

### 3.1. Morphology and Structure

**Figure 2:**
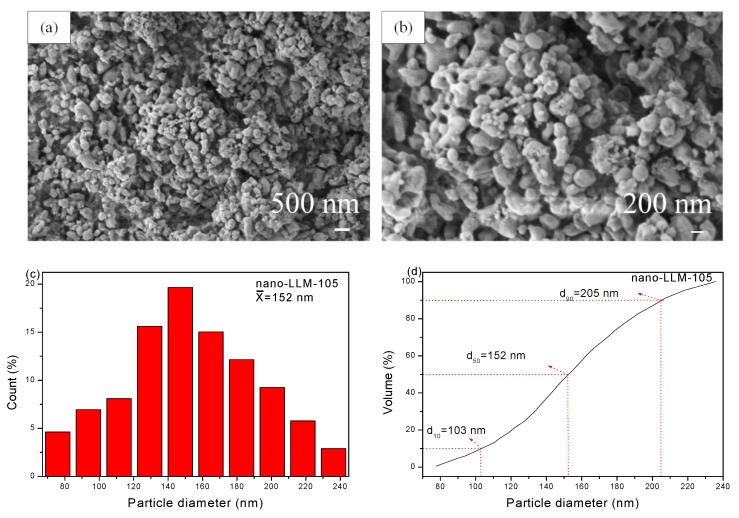
(**a**,**b**) SEM image of LLM-105 nanoparticles; (**c**,**d**) diameter distribution of the LLM-105 nanoparticles.

**Figure 3:**
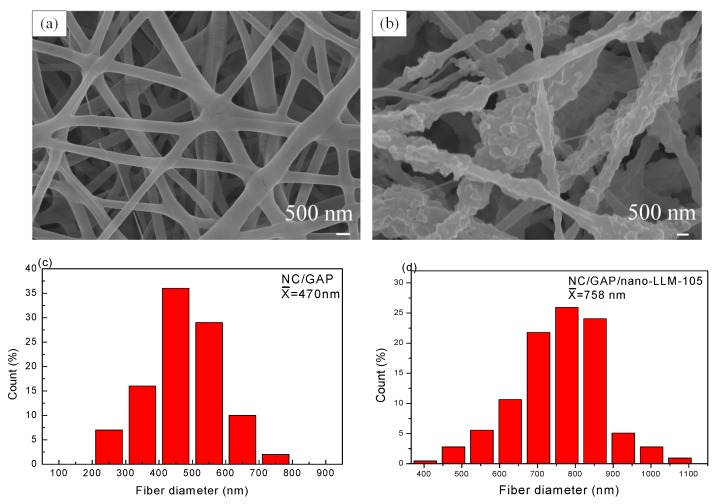

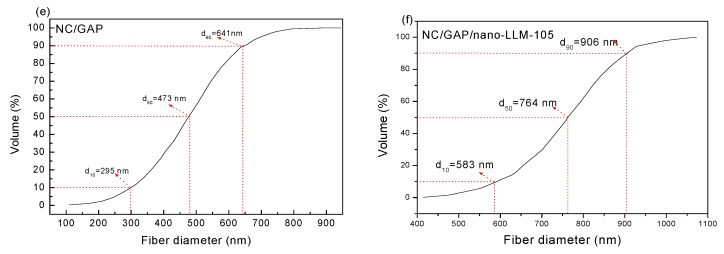
Images and diameter distributions of samples: (**a**) for NC/GAP; (**b**) for NC/GAP/nano-LLM-105; (**c–f**) the diameter distribution.

Figure 2a,b reveals that there are some weaker agglomerates rather than hard agglomerates for LLM-105 nanoparticles, and there are no bridge between the particles. The particle diameter distribution is obtained by measuring a diameter of ~100 particles, and the results are displayed in Figure 2c–d. We acquired the volume curve by integrating the frequency curve. The mean diameter calculated from the frequency curve is 152 nm, which is same as the median diameter (d_50_ = 152 nm) calculated from the volume curve. For the SEM images of NC/GAP and NC/GAP/nano-LLM-105 (Figure 3a,b), it is clearly observed that both of the two nanofibers reveal 3D reticulate structures. The surface of the NC/GAP nanofiber is smooth and uniform. On the contrary, the surface of the NC/GAP/nano-LLM-105 nanofiber is rough and uneven. The difference in morphology is primarily caused by two factors. The addition of LLM-105 nanoparticles results in an inhomogeneity of precursor solution. Furthermore, partial LLM-105 nanoparticles are agglomerated during the electrospinning process. From Figure 3c–f, for NC/GAP nanofibers, the mean diameter and median diameter are 469 nm and 478 nm, respectively. By comparison, the mean diameter and median diameter of NC/GAP/nano-LLM-105 are 758 nm and 764 nm, respectively. It is apparent that the diameter of NC/GAP/nano-LLM-105 is larger than that of the NC/GAP nanofibers. The difference in mean diameter of these two nanofibers is due to the fact that the LLM-105 nanoparticles are loaded onto the surface of NC/GAP.

**Figure 4:**
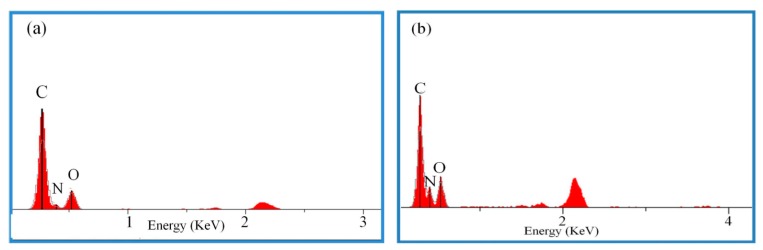
EDS spectra of samples: (**a**) for NC/GAP; (**b**) for NC/GAP/nano-LLM-105.

EDS analyses were performed to probe the surface elements of the nanofibers; the results are exhibited in Figure 4. From Figure 4a,b, the peaks at about 2 Kv belonged to the gold element sprayed during the test. There were only O, C, and N elements that were presented on the surfaces of those two fibers, illustrating that impurities were not introduced in the process of ball milling and electrospinning. The theoretical elemental contents are tabulated in Table 1. After the addition of the LLM-105 nanoparticles, the O content hardly varied, the N content appreciably increased, and the C content declined. 

**Figure 5:**
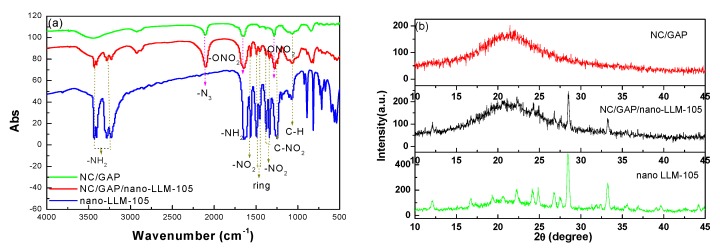
IR spectra (**a**) and XRD patterns (**b**) of NC/GAP, nano-LLM-105 and NC/GAP/nano-LLM-105.

The IR spectra of NC/GAP, NC/GAP/nano-LLM-105, and nano-LLM-105 are contrasted in Figure 5a. For NC/GAP/nano-LLM-105, the peaks at 3437, 3405, 3285 3233, and 1648 cm^−1^ respectively indicated symmetric, anti-symmetric stretching vibrations and deformation vibrations of –NH_2_ in nano-LLM-105; two strong absorption peaks located at 1480 and 1448 cm^−1^ corresponded to the stretching vibrations of the C=C skeleton in the ring of nano-LLM-105; the peak at 1577 cm^−1^ indicated anti-symmetric stretching vibrations of –NO_2_ in nano-LLM-105; the peaks at 1351 cm^−1^ and 1383 cm^−1^ corresponded to symmetric stretching vibrations of –NO_2_ in nano-LLM-105 [27]; the peak at 2101 cm^−1^ was ascribed to the stretching vibrations of –N_3_ from the GAP that was present [28]; the peaks at 1280 and 1648 cm^−1^ reflected the symmetric and anti-symmetric stretching vibrations of -ONO_2_ in NC, respectively [29]; the peak at 1075 cm^−1^ corresponded to the out-of-plane bending vibrations of C–H. Hence, the functional groups for NC/GAP/nano-LLM-105 were in accord with nano-LLM-105 and NC/GAP, indicating that the LLM-105 nanoparticles were well-combined with NC/GAP. These peaks were the same as the published report. Overall, the molecular structures of the nano-LLM-105 and NC/GAP do not alter in the process of electrospinning. There were no new groups generated, indicating that NC, GAP, and nano-LLM-105 do not react with each other. Figure 5b shows the XRD patterns of samples. There were two main peaks at 28.4 and 33.2° in pattern of nano-LLM-105 [10]. Also, there were no diffraction peaks in the pattern of NC/GAP. This is because LLM-105 is a type of crystal, and NC/GAP is a type of polymer. The peak positions of the LLM-105 nanoparticles were in line with those of the NC/GAP/nano-LLM-105 nanofibers, which means that the crystal phase of the LLM-105 nanoparticles does not transform by electrospinning. This is a superior feature of the electrospinning compared with recrystallization to prepare energetic materials. For the recrystallization method, if the solvent is not properly selected, the crystal phase is liable to transform. Song prepared 1,3,5,7-Tetranittro-1,3,5,7-tetrazocane (HMX) by solvent/non-solvent method, and the crystal phase of HMX changed from β-HMX to γ-HMX [30]. In this work, acetone is chosen as the solvent, and the LLM-105 nanoparticles are suspended in it, which avoids the recrystallization of the LLM-105. 

**Figure 6:**
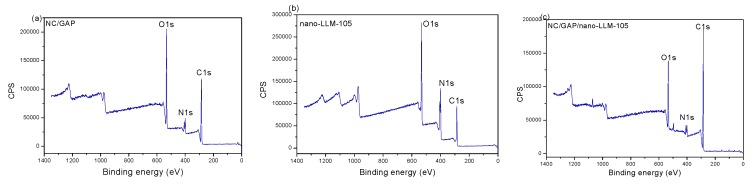

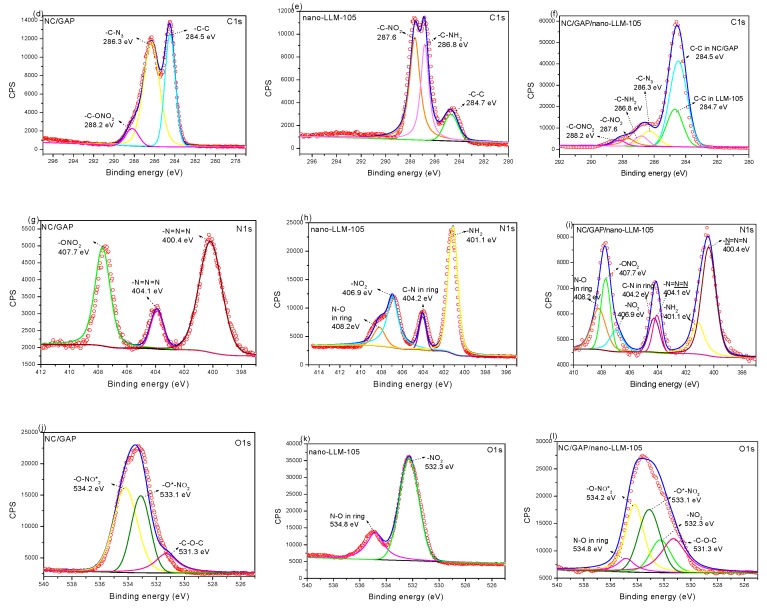
(**a**–**c**) XPS spectra of NC/GAP, nano-LLM-105 and NC/GAP/nano-LLM-105; high resolution XPS spectra (**d**–**f**) for C 1s of samples; for (**g**–**i**) N 1s of samples and for (**j**–**l**) O 1s of samples.

The XPS spectra of NC/GAP, nano-LLM-105, and NC/GAP/nano-LLM-105 are displayed in Figure 6. From Figure 6a–c, typical signals of C, N, and O were clearly detected. For NC/GAP/nano-LLM-105, the O1s spectra presented five features with binding energies of 531.3 eV, 532.3 eV, 533.1 eV, 534.2 eV, and 534.8 eV; the peaks at 532.3 eV and 534.8 eV were ascribed to -NO_2_ and N-O in the ring of LLM-105; the peaks at 533.1 eV and 534.2 eV belonged to -O*-NO_2_ and -O-NO*_2_ in NC; the peak at 531.3 eV was related to the -C-O-C group of NC and GAP [31]. For the C1s spectrum of NC/GAP/nano-LLM-105, the peak was fitted to six peaks. The peaks located at 284.5 eV, 286.3 eV, and 288.2 eV were assigned to -C-C, C-N_3_, and -C-ONO_2_ of GAP and NC. The peaks at 284.7 eV, 286.8 eV, and 287.6 eV corresponded to -C-C, -C-NH_2_, and -C-NO_2_ in nano-LLM-105 [32]. The XPS spectrum of N1s consisted of seven peaks at 400.4 eV, 401.1 eV, 404.1 eV, 404.2 eV, 406.9 eV, 407.7 eV, and 408.2 eV, which corresponded respectively to –N=N=N, –NH_2_, –N=N=N, C-N in ring, -NO_2_, –ONO_2_, and N-O in the ring [28]. The -N_3_ and –ONO_2_ groups were ascribed to NC and GAP, respectively. Finally, the groups of C-N in ring, N-O in ring, –NH_2_ and -NO_2_ belong to LLM-105. Hence, we infer the existence of LLM-105 nanoparticles on the surface of NC/GAP, and there are no new chemical bonds being produced on the surface of the nanofibers.

**Figure 7:**
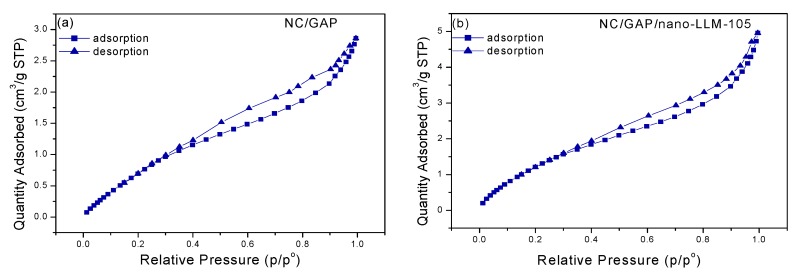
The BET data of samples: (**a**) NC/GAP and (**b**) NC/GAP/nano-LLM-105 nanofiber.

The nitrogen adsorption–desorption isotherms of NC/GAP and NC/GAP/nano-LLM-105 are displayed in Figure 7. The specific surface areas, pore volumes, and pore sizes of the samples are shown in Table 2. The isotherms are considered as class IV (H3-type hysteresis loop), indicating that the prepared nanofibers were mesoporous materials. At a low p/p^o^, there is the first steep portion of the isotherm, as the p/p^o^ increases, adsorption of multiple layers begins. In the multi-layer adsorption process, capillary condensation a common accompaniment (IV isotherms). Capillary condensation and capillary evaporation generally do not occur at identical p/p^o^, resulting in the generation of hysteresis loops. The specific surface areas of the NC/GAP/nano-LLM-105 and NC/GAP nanofibers were 6.0545 and 4.3573, respectively. The higher surface area value of NC/GAP/nano-LLM-105 is ascribed it having a rough surface. Compared to the energetic composite prepared by other methods, energetic nanofibers have a larger specific surface area. For example, the specific surface area of NC/GAP/CL-20 when prepared by a sol-gel-supercritical method, is 2.7 [33].

### 3.2. Thermal Analysis

**Figure 8:**
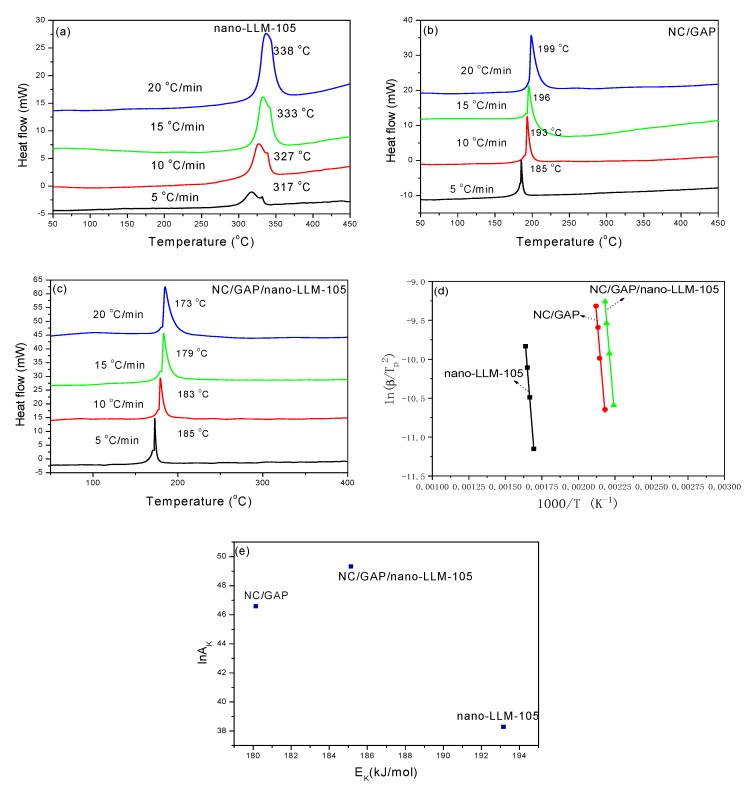
Thermal analyses of the samples. (**a**–**c**) DSC thermograms of samples collected at different heating rates. (**d**) Kissinger plots of ln(β/Tp^2^) to 1000/Tp; (**e**) for the kinetic compensation effect.

The DSC thermograms of samples collected at different heating rates are displayed in Figure 8a–c. The kinetic and thermodynamic parameters for thermal decomposition are calculated with the DSC data; the results are displayed in Table 3. For all samples, the exothermic peak temperature increases with the increase of heating rate. For NC/GAP/nano-LLM-105, there was only one exothermic peak, demonstrating that NC, GAP, and nano-LLM-105 decompose synchronously. In addition, the exothermic peak temperature of NC/GAP/nano-LLM-105 is slightly lower than that of NC/GAP, and it is markedly lower than those of the LLM-105 nanoparticles. This manifests that the thermolysis of NC, LLM-105 nanoparticles, and GAP cooperate with each other. 

The activation energy (*E_K_*), pre-exponential factor (*A_K_*), and rate constant (*k*), are calculated by the Kissinger equation (Equation (1)) [29] and the Arrhenius equation (Equation (2)) [34]. The *E_K_* of NC/GAP/nano-LLM-105 (185.139 kJ·mol^−1^) is lower than that of the LLM-105 nanoparticles and is higher than that of NC/GAP. The *k* of NC/GAP/nano-LLM-105 is higher than the *k* of the NC/GAP and LLM-105 nanoparticles, implying that NC/GAP/nano-LLM-105 has a higher decomposition rate.

(1) $$\ln\frac{\beta }{{T_{p}}^{2}}=\ln\frac{R\cdot A_{K}}{E_{K}}-\frac{E_{K}}{R}\cdot \frac{1}{T_{p}}$$

(2) $$k=A_{K}\cdot Exp\left(-\frac{E_{K}}{T_{p}\cdot R}\right)$$

(3) $$A_{K}\exp\left(-\frac{E_{K}}{RT_{P}}\right)=\frac{K_{B}T_{P}}{h}\exp\left(-\frac{\Delta G^{\neq }}{RT_{P}}\right)$$

(4) $$\Delta H^{\neq }=E_{K}-RT_{P}$$

(5) $$\Delta G^{\neq }=\Delta H^{\neq }-T_{P}\Delta S^{\neq }$$

where *T_p_* is the peak temperature in the DSC trace, with a heating rate of 15 °C·min^−1^; *K_B_* and *h* are the Boltzmann (*K_B_* = 1.381 × 10^−23^ J/K) and Planck constants (h = 6.626 × 10^−34^ J/s), respectively; β is the heating rate.

The thermal decomposition of the energetic molecules originates from the activation and rupture of the weakest bond, which is quite significant for the decomposition process. As the temperature of the explosive increases, the molecular thermal motion is enhanced. When the temperature attains a critical point, the weakest bond will be stretched. Subsequently, a rupture occurs. This activation process could be described by the parameters of activation enthalpy (Δ*H^≠^*), activation free energy (Δ*G^≠^*), and activation entropy (Δ*S^≠^*), as calculated by Equations (3)–(5) [27]. Δ*H^≠^* is the energy that the molecules absorb to transform from a common state to an activated state. Compared with nano-LLM-105, NC/GAP/nano-LLM-105 needs a lower level of energy to be activated. Δ*G^≠^* is the chemical potential of the activation course. For all of these samples, the values of Δ*G^≠^* are positive numbers, indicating that none of the activation courses proceed spontaneously [28]. Figure 8e shows a kinetic compensation effect during the thermolysis of NC/GAP, nano-LLM-105, and NC/GAP/nano-LLM-105. The three points do not present a linear relationship, which means that the three samples have disparate kinetic mechanisms of decomposition. In addition, Wang prepared 1,3,5,7-tetranittro-1,3,5,7-tetrazocane/nitrocellulose (HMX/NC) and NC/GAP/CL-20 by the sol-gel method. Similarly, the samples do not have just one exothermic peak, indicating that thermal decomposition for them is carried out in multiple steps. Moreover, *E_K_* of HMX/NC iii and NC/GAP/CL-20 i are 277.68 kJ·mol^−1^ and 296 kJ·mol^−1^, respectively, which are significantly higher than the *E_K_* of NC/GAP/nano-LLM-105 [29,33]. This is because, compared with the nanofibers that are prepared by electrospinning, the components of an energetic composite prepared by the sol-gel method cannot be tightly combined with each other, and there is a lower mutual promotion of the components for thermal decomposition.

**Figure 9:**
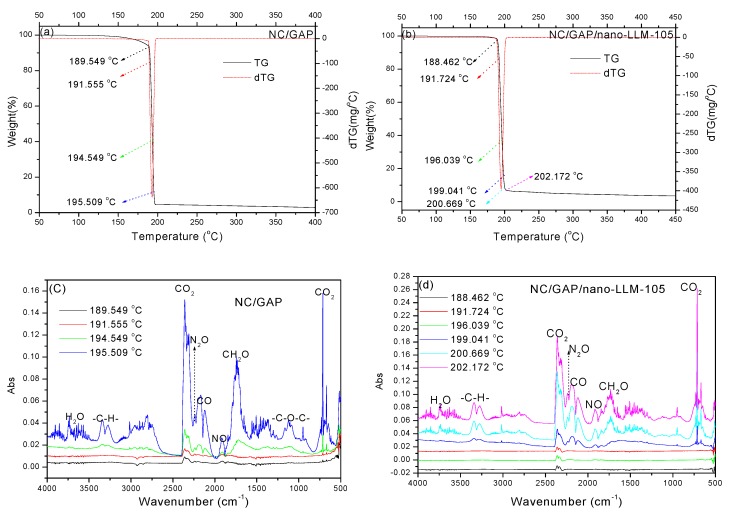
TG-IR analysis of samples: (**a**,**b**) TG and DTG curves; (**c**,**d**) IR spectra of the decomposition products at different temperatures.

The products for the thermal decomposition of NC/GAP/nano-LLM-105 and NC/GAP were investigated by TG-IR. The TG and DTG curves are displayed in Figure 9a,b, and the IR spectra at different temperatures are shown in Figure 9c,d. For NC/GAP, the decomposition began at 177 °C, and the decomposition rate reached its maximum at 193.11 °C. The decomposition almost finished at 196.6 °C. For NC/GAP/nano-LLM-105, the initial decomposition temperature rose to 188.65 °C. Composite nanofibers decomposed at the fastest rate at 195.07 °C. Also, the decomposition was generally accomplished at 201.04 °C. This means that the decomposition of NC/GAP/nano-LLM-105 is more concentrated. The IR spectrum, indicated that the main products were diverse. The main peaks and attributions are listed in Table 4. For clarity, the curves are vertically offset in Figure 9c,d. The strong peaks at 2309–2360 cm^−1^ indicated the presence of much CO_2_ gas. The peaks in 2113–2199 cm^−1^ represented the appearance of CO. The weak peak located at 2239 cm^−1^ manifested the existence of a very low amount of N_2_O gas. The existence of NO explicates the peaks that were located at 1901–1924 cm^−1^. Moreover, the peaks in 3271–3379 cm^−1^ and 1691–1788 cm^−1^ corresponded to the fragments of -CH and -CH_2_O, respectively. The –N_3_ is the energetic group of GAP, which is decomposed to N_2_. In reality, N_2_ is a nonpolar molecule, and it cannot be probed by IR. For those two nanofibers, the positions of the main peaks are mainly identical. The only difference is the generation of -C-O-C- fragments for NC/GAP nanofibers. However, there are no -C-O-C- fragments for NC/GAP/nano-LLM-105. In addition, compared with NC/GAP, the peak intensities of the-CH_2_O fragments decreased significantly. This indicates that for NC/GAP/nano-LLM-105, the fragments of -CH_2_O and -C-O-C- further reacted to generate CO_2_, CO, and H_2_O. This is because the interposition of the LLM-105 nanoparticles in NC/GAP improved the oxygen balance.

### 3.3. Energetic Performance and Sensitivities

**Figure 10:**
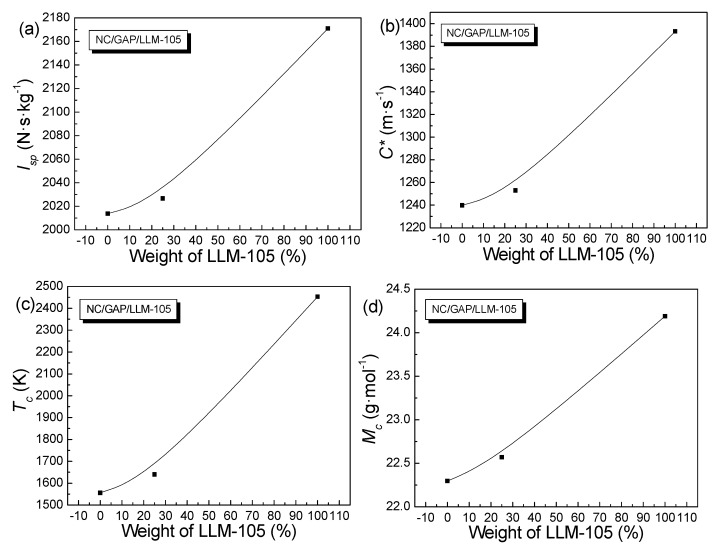
Energy performances of NC/GAP/LLM-105 nanofibers as a function of the weight percentage of LLM-105: (**a**) for *I_sp_*; (**b**) for C*; (**c**) for *T_c_* and (**d**) for *M_c_*.

**Figure 11:**
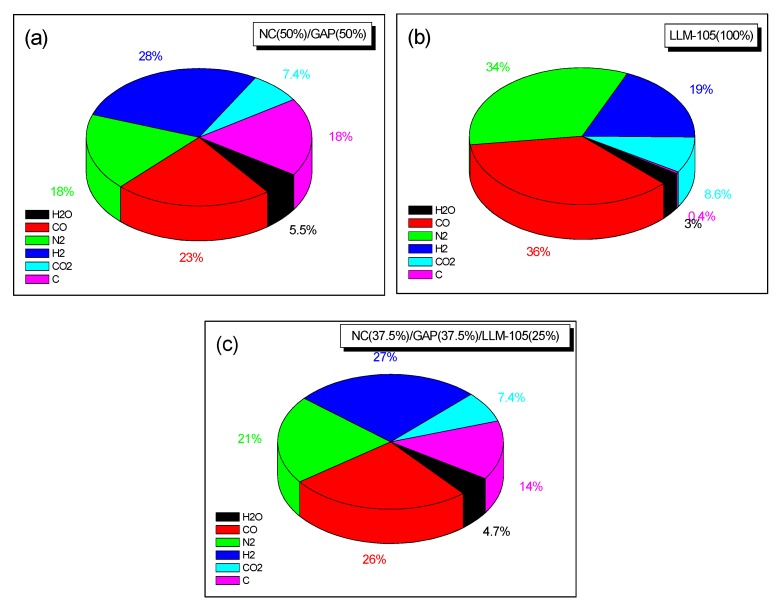
(**a**–**c**) Combustion products and their molar ratios for NC/GAP/LLM-105 nanofibers. The results in Figure 11 were calculated by the means of the ProPep 3.0 software under conditions of *P_c_*/*P_e_* = 70/1 (*P_e_* = 0.1 MPa) and *T*_0_ = 298 K.

To further explore the energy properties of NC/GAP/nano-LLM-105, their energy performances and impact sensitivities were evaluated. The standard specific impulse (*I_sp_*), characteristic velocity (C*), combustion chamber temperature (*T_c_*), and average molecular weight (*M_c_*) were calculated, the results are listed in Table 5. The functional relationships between the energy performances of NC/GAP/LLM-105 nanofibers and the weight percentage of LLM-105 are shown in Figure 10. For nano-LLM-105, NC/GAP, and NC/GAP/nano-LLM-105, the standard deviations of *H*_50_ were 4.5, 4.0 and 4.4, respectively. The impact sensitivities of the samples were tested, and the results are displayed in Table 4. Furthermore, the combustion products and their mass molar ratios were calculated, and the results are shown in Figure 11. The feature height (*H*_50_) for NC/GAP/nano-LLM-105 was significantly higher than *H*_50_ for NC/GAP, and it was a little lower than nano-LLM-105. This indicates that the impact sensitivity of NC/GAP/nano-LLM-105 was distinctly lower than that of NC/GAP, and slightly higher than that of nano-LLM-105. For energy performance, the standard specific impulse (*I_sp_*) of NC/GAP was 2013.8 N·s·kg^−1^. For NC/GAP/nano-LLM-105, the *I_sp_* value increased to 2032.4 N·s·kg^−1^. This was not attributed to the high energy of LLM-105, but it was rather due to the higher formation enthalpy (Δ*H_f_*) and C/H values of LLM-105, in contrast to those of NC/GAP. The formation enthalpy of LLM-105 (−13 kJ·mol^−1^) was observably larger than that of NC/GAP (−294.6 kJ·mol^−1^). Therefore, after LLM-105 was introduced into NC/GAP, the energy performance was dramatically improved. The value of oxygen balance (OB_CO2_) was a crucial element for assessing the energy performance. As the OB_CO2_ increased, the energy performance is enhanced. The OB_CO2_ of LLM-105 (−37.03) is higher than that of NC/GAP (−76.1). Hence, the introduction of LLM-105 is in favor of improvement for the energy performance. Additionally, the C/H mass ratios of nano-LLM-105 and NC/GAP are 12 and 8.24, respectively. We infer that the higher OB_CO2_ and C/H are beneficial to enhancement under combustion temperature (*T_c_*) [33,35]. *T_c_* represents the chemical energy storage of an energetic formulation, which is proportional to the explosive heat of propellants. Figure 10c shows that the value of *T_c_* increases as the weight percentage of LLM-105 increases. The energy performance is mainly determined by the heat released from combustion, and the energy conversion efficiency. The latter is related to the hydrogen content in the molecules. The decrease of hydrogen content leads to a decrease of H_2_ content. Therefore, the average molecular weight (*M_c_*) increases, which is disadvantageous to energy conversion efficiency. From Figure 11, for NC/GAP, H_2_ accounts for 28% in combustion products, and the H_2_ proportion of LLM-105 is 19%. Hence, the *M_c_* of LLM-105 is higher than that of NC/GAP in Table 4. Figure 10d shows that the value of *M_c_* increases as the weight percent of LLM-105 increases. In this case, although *M_c_* of LLM-105 is higher than that of NC/GAP, the *I_sp_* of LLM-105 is still significantly higher than *I_sp_* of NC/GAP. This is because the negative effect of low energy conversion efficiency is offset by the high storage of chemical energy. The higher chemical energy storage of LLM-105 is attributed to its higher C/H mass ratio, OB_CO2_, and the formation enthalpy. Therefore, the interposition of LLM-105 improves the energy performance of NC/GAP.

## 4. Conclusions

The NC/GAP/nano-LLM-105 composite nanofiber with a large specific surface area was prepared by an electrospinning technique. Compared with NC/GAP and LLM-105 nanoparticles, NC/GAP/nano-LLM-105 nanofibers have lower decomposition temperature and distinctly higher decomposition rate. The activation energy of NC/GAP/nano-LLM-105 for thermolysis is lower than that of LLM-105 nanoparticles. These indicate that NC/GAP/nano-LLM-105 decompose relatively easily and violently. If it is used in solid rocket propellant systems, it will decompose first, and then induce the decomposition of other components. 

The *I_sp_* and *T_c_* of NC/GAP/nano-LLM-105 are higher than those of NC/GAP, which means that NC/GAP/nano-LLM-105 possesses a distinguished energy performance. In addition to the energy performance, safety is another rather crucial factor for energetic materials. The impact sensitivity of NC/GAP/nano-LLM-105 is signally lower than that of NC/GAP. Hence, it has a good safety performance rating. NC/GAP/nano-LLM-105 possess both energy performance and low impact sensitivity. Therefore, this composite nanofiber has enormous potential in the field of solid rocket-propellant systems. This versatile preparation method may provide a concept for synthesizing energetic nanocomposites. 

## Figures and Tables

**Table 1:** Theoretical elemental contents from EDS analyses.

Sample	C Content (%)	N Content (%)	O Content (%)
NC/GAP	31.45	27.5	37.2
NC/GAP/nano-LLM-105	29.14	30.20	37.16

**Table 2:** BET surface area and the pore structure parameters of the nanofibers.

Samples	BET Surface Area (m^2^·g^−1^)	Pore Volume (cm^3^·g^−1^)	Pore Size (nm)
NC/GAP	4.3573	0.004422	4.05911
NC/GAP/nano-LLM-105	6.0545	0.007664	5.06352

**Table 3:** Thermodynamics and kinetics deduced from DSC traces.

Samples	Tp (K)	Thermodynamics			Kinetics		
		Δ*H^≠^* (kJ·mol^−1^)	Δ*G^≠^* (kJ·mol^−1^)	Δ*S^≠^* (J·mol^−1^·K^−1^)	*E_K_* (kJ·mol^−1^)	ln*A_K_*	*k* (s^−1^)
Nano-LLM-105	606	188	152	59	193	38	1.0
NC/GAP	468	176	115	130	180	47	1.4
NC/GAP/Nano-LLM-105	456	181	111	153	185	49	1.7

**Table 4:** Peaks and attributions.

Samples	Peaks and Attribution (cm^−1^)							
	3739–3560	2309–2360	2339	2113–2199	1901–1924	3271–3379	1691–1788	1077–1130
NC/GAP	H_2_O	CO_2_	N_2_O	CO	NO	-C-H	-CH_2_O	C-O-C
NC/GAP/nano-LLM-105	H_2_O	CO_2_	N_2_O	CO	NO	-C-H	-CH_2_O	no

**Table 5:** Impact sensitivity and energy performance of the samples.

Samples	Impact Sensitivity	Energy Performance			
	H_50_ (cm)	I_sp_ (N·s·kg^−1^)	C* (m·s^−1^)	T_c_ (K)	M_c_ (g·mol^−1^)
NC(50%)/GAP(50%)	60	2014	1240	1556	22
LLM-105(100%)	113	2171	1393	2453	24
NC(37.5%)/GAP(37.5%)/LLM-105(25%)	78	2027	1253	1640	23

*I_sp_* is the standard specific impulse; C* is the characteristic speed; *T_c_* is the combustion chamber temperature; *M_c_* is the average molecular weight of the combustion products. All of the parameters were calculated by the means of software ProPep 3.0 at condition of *P_c_*/*P_e_* = 70/1 (*P_e_* = 0.1 MPa) and *T*_0_ = 298 K.
